# A novel 3D-printed simulator for hands-on training in orthognathic surgery

**DOI:** 10.1186/s40902-025-00462-z

**Published:** 2025-05-23

**Authors:** Lukas Benedikt Seifert, Christopher Groepper, Rosa Rohin, Daniel Thiem, Philipp Becker, Florian Markus Thieringer, Robert Alexander Sader

**Affiliations:** 1 University Hospital of Basel, Basel, Switzerland; 2 Department of Oral-, Craniomaxillofacial and Facial Plastic Surgery, Goethe University Frankfurt Germany, 60596 Frankfurt, Germany; 3 University Medical Center of the Johannes Gutenberg University Mainz, Mainz, Germany; 4 Bundeswehrzentralkrankenhaus Koblenz, Koblenz, Germany

**Keywords:** 3D printing, Orthognathic surgery, Surgical education

## Abstract

**Background:**

Orthognathic surgery addresses skeletal dysgnathias, enhancing both function and aesthetics. Despite its benefits, potential complications underscore the need for well-trained surgeons. 3D-printed anatomical models, a product of additive manufacturing, aid surgical education, especially for young surgeons lacking on-the-job training. This study focuses on an economically designed 3D-printed simulator for orthognathic surgery training.

**Results:**

Evaluation from 31 participants of two orthognatic surgery workshops using the 3D-printed simulator highlighted positive assessments for realism (7.16 ± 2.03/10) and usefulness in training for specific procedures. While commended for simulating limited oral cavity movements and providing a realistic general simulation, soft tissue realism (4.51 ± 2.66/10) suggested room for improvement. Notably, the simulator demonstrated outstanding cost-efficiency (€ 181.55), with reusable components.

**Conclusions:**

The 3D-printed simulator offers a realistic, cost-effective tool for orthognathic surgery training, despite soft tissue realism limitations. The study anticipates further enhancements in 3D-printing technology to address these aspects and advance future iterations.

## Background

Dysgnathias are characterised as congenital, inherited or acquired anomalies of the teeth and jaws that often lead to functional impairments and an inharmonious appearance. The aim of orthognathic surgery is to treat skeletal dysgnathias in order to improve both the function of the stomatognathic system as well as the facial profile and, thus, aesthetics [1]. Even though, today orthognathic surgery is considered a standard procedure in oral and maxillofacial surgery it is still a highly technically sensitive operation which requires a thorough spatial understanding of the complex anatomy in the head and jaw region and years of training in order to prevent intra-operative complications like major bleedings [2], damaging of the neuro-vascular bundle [3, 4] or a bad split of the mandible [5, 6]. In order to gain the required expertise, resident surgeons typically undergo various stages of learning, starting with observation and progressing to providing surgical assistance, until they eventually conduct specific parts of surgeries independently while being supervised. This traditional “See One, Do One” approach has been a cornerstone of surgical education since its introduction by William Halsted in 1890; however, many argue that one can not safely perform a surgery only by observing it once [7]. Moreover, the increasing economisation in health services as well as ethical restrictions offer less opportunities for a on-the-job training of young surgeons [8].

Given this context, modern simulation training, originating from the aviation industry [9], has gained significant traction in surgical education. This approach is growing in popularity because it allows trainees the opportunity to make errors without jeopardizing the patients’ health, leaves out the added stress of performing surgery on a live patient and reduces surgical errors which ultimately helps to increase patient safety [10]. In recent years a variety of possible simulators for the training of surgical procedures in Oral and Maxillofacial Surgery have been decribed ranging from the use of animal cadavers [11] over human anatomical specimen [12] to high fidelity virtual simulators [13]. Animal cadavers like pig jaws are relatively affordable to purchase and often used in the training of surgical procedures; howewer, when it comes to mimicing the human anatomy, they often lack realism. Human anatomical specimen are very realistic but ethical and financial restrictions strongly limit their use. High fidelity simulators are more frequently used but also cost-intensive which also limits their use especially in non-industrialized countries.

Additive manufacturing also known as 3D-printing (3DP) was first introduced in 1986 by Charles Hull [14] and has since undergone an explosive increase in use in many industries including the medical sector. Today, there are a multitude of open-source software solutions and affordable 3D-printers that allow clinicians to easily print a physical model obtained from (cone-beam) CT or MRI data sets. Clinically used applications for such models include preoperative planning and visualization, low cost implant production via moulding [15], pre-bending of osteosynthesis material [16] or patient education [17]. Due to their capacity to replicate any given pathology in a physical model 3D-printed simulator also have extensively been used in surgical education over the last years [18, 19]. Due to the complexity of the procedure there only have been a few working groups that tried to build a surgical simulator for the training of orthognathic surgery. Bertin et al. used a 3D-printed model of a mandible to train residents in the performance of a bilateral sagittal split osteotomy [20]. Likewise, Yoshida et al. used 3D-printed models of a maxilla and a mandible to train undergraduate dental students in the performance of a LeFort I osteotomy and a bilateral sagittal split osteotomy [21]. Even though both studies used 3D-printed models as simulators, apects like the soft-tissues, the limited space of movements in the oral cavity and the positional relation and articulation of the maxilla and mandible were not considered in these studies.

Hence, the aim of the current study was to develop a 3D-printed simulator for the training in orthognathic surgery and evaluate its use regarding surgical realism and cost-effectiveness.

## Material and methods

### Production of the replaceable facial skull model

**Fig. 1:**
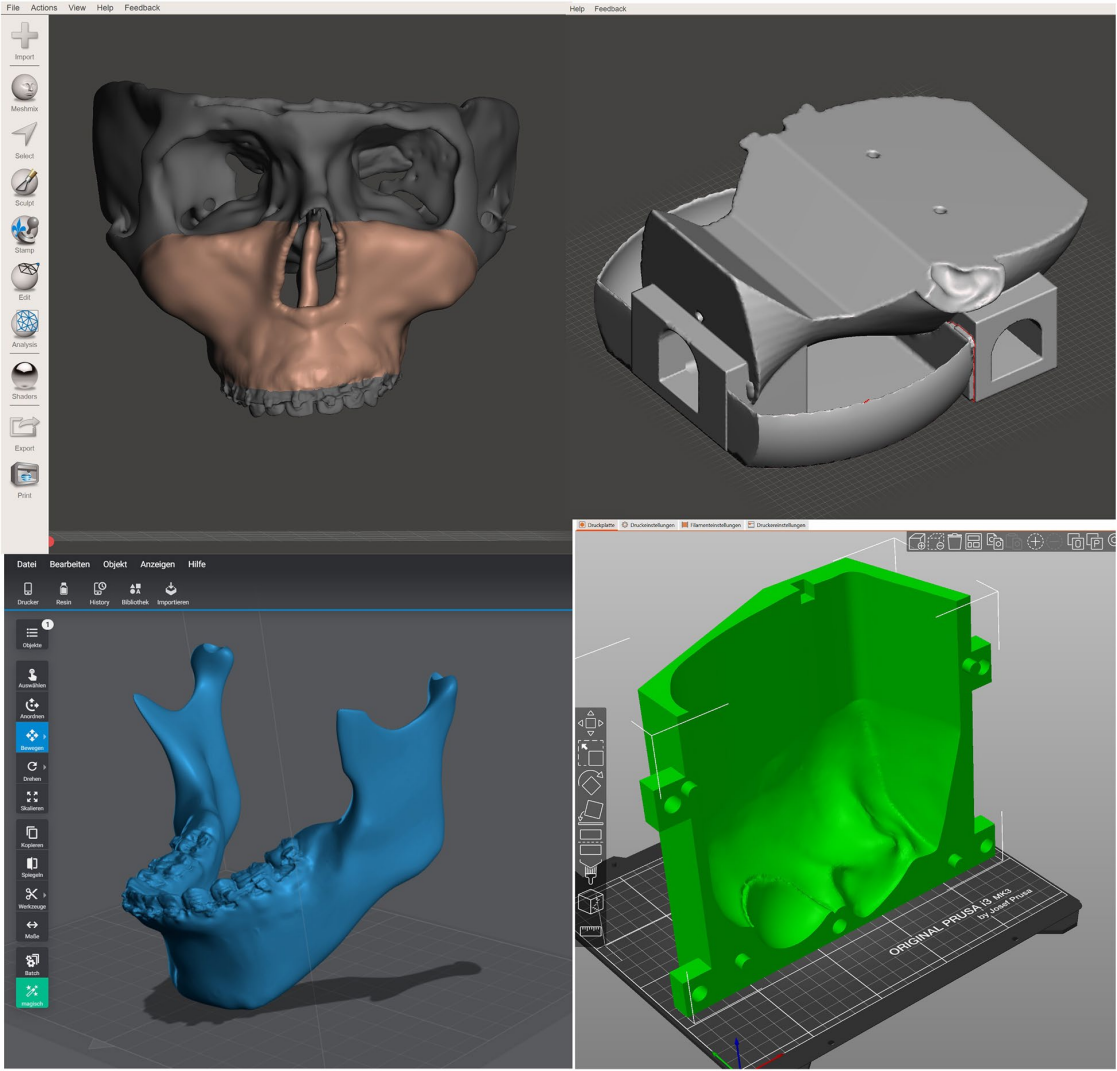
Computer-assisted design of the simulators components

A anonymized CBCT data set of a male patient with a skelletal dysgnathia (maxillary prognathism, mandibular retrognathism, frontal open bite) was used for the production of the replaceable facial skull model. To convert the existing DICOM (Digital Imaging and Communications in Medicine) data sets into an.stl (Standard Triangle Language) format file the freely available software 3DSlicer [22] was used. Further modifications on the resulting.stl files were made in the also freely available software Meshmixer (Autodesk inc., USA). The surface was post-processed, artifacts were removed and the skull and maxilla were separated from the mandible (Fig. 1). Small recesses were added to the condylar process to attach ball-joint magnets later on. Likewise, corresponding recesses were designed in the area of the mandibular fossa. The virtual skull was reduced behind the temporomandibular joints in the coronal layer around the back of the head. Instead, a flat holding surface was constructed, on which two recesses were also configured for later fixation with screws.

A virtual gingival mask was designed using the upper and lower jaw surfaces. To generate a printed, accurately fitting gingival mask, the surface to be covered with was marked in the Meshmixer software and separated from the rest of the data set. This new surface was then extruded outwards by 2.5 mm, thus generating a new surface that is an exact fit to the facial skull and represents a 2.5-mm-thick virtual gingiva (Fig. 1).

**Fig. 2:**
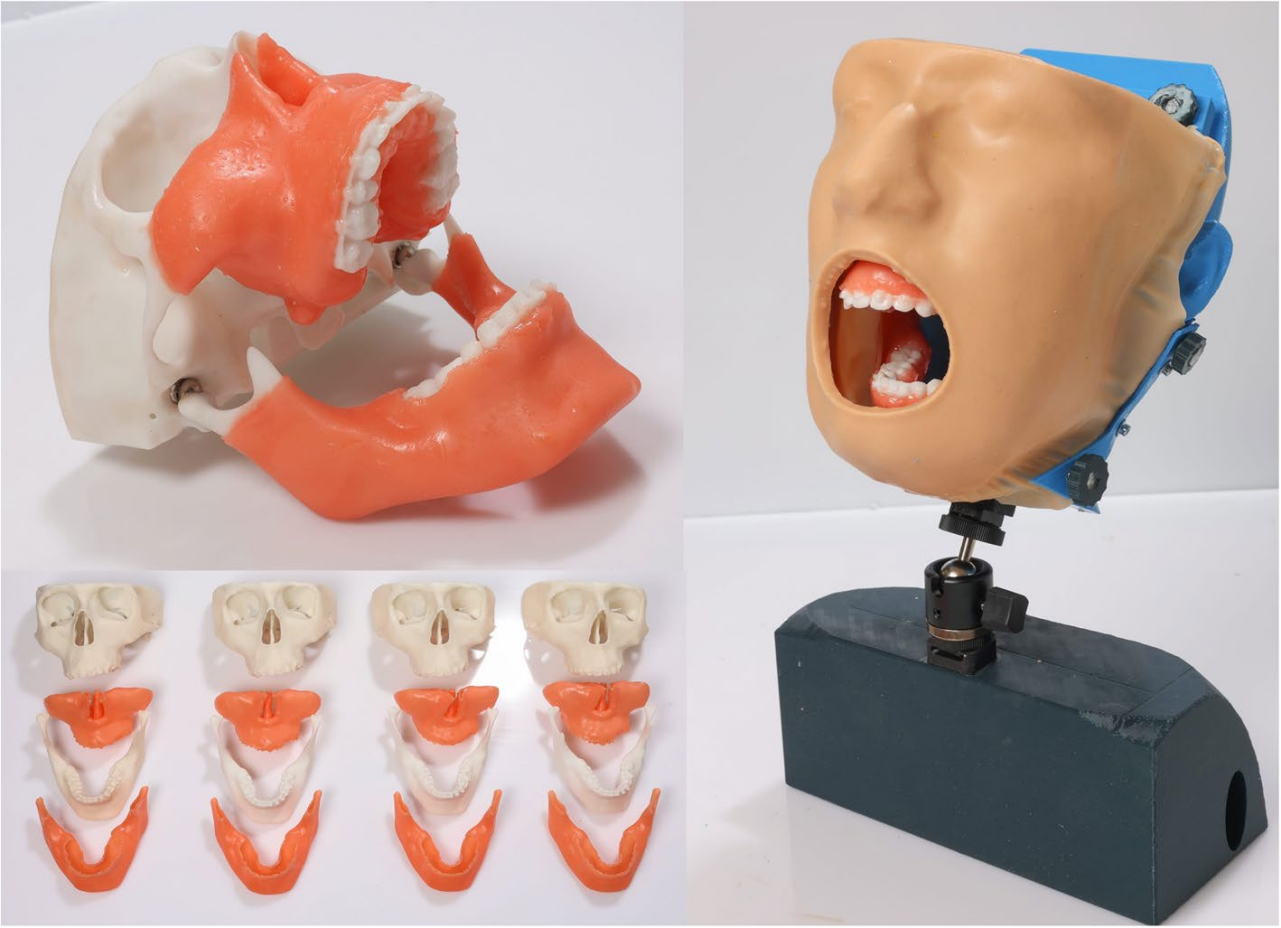
Final 3D-printed simulator with attached gingival mask, table/chair mounting and silicone face mask

The resulting.stl-files of the skull model and gingival masks were prepared for 3D printing with freely available Chitubox software (Chitubox, Guangdong, China) with the following settings for the skull model: layer thickness 0.1 mm and exposure time 4 s. For the gingival masks, the layer thickness was 0.2 mm and the exposure time was 10 s. The components were then printed using the digital light processing (DLP) technique on a Anycubic Photon Mono X Desktop 3D-printer (Anycubic, Shenzhen, China) using the white Anycubic rapid resin (Anycubic, Shenzhen, China) for the skull model and the Resione F80 elastic resin in gum color (Resione, Guangdong Province, China) for the gingival masks. After each print, the models were post-processed as follows: removal of all support structures, cleaning of the models with 99%isopropanol and curing with UV light in the Anycubic Wash and Cure unit (Anycubic, Shenzhen, China) for 6 min. A threaded pin was glued to the mandibular models in the area of the condylar process in order to screw in the reusable ball joint magnet (MagnaC GmbH, Wendlingen, Germany). The cup of the reuseable ball joint magnet was inserted at the maxillary recesses in the area of the mandibular fossa. The gingiva was finally attached to the bone models by polymerising liquid resin (Fig. 2).

### Production of the reusable bench top phantom head and facial mask

A virtual phantom head dummy model was imported from the Blender software databank [23] into the Meshmixer software (Autodesk Inc., USA). The model was then reduced at the back of the dummy’s head and a virtual basin for splash water was added. Moreover, a total of five recesses were added, two for the later screw fixation of the replaceable bone models and three more for attaching the silicone face mask (Fig. 1).

The virtual face mask based on the DICOM datasets and facial counters of the original patient was also generated in the Slicer software [22] and then modified in the Meshmixer software (Autodesk Inc., USA). In order to be able to silicone cast the face mask, a positive mould and a negative mould were generated using the existing data set (Fig. 1).

Both models were finally printed using an Prusa MK3 S + (Prusa Research a.s., Czech republic) using black Prusament PLA (Prusa Research a.s., Czech republic).

After printing, all support structures were removed and threaded inserts were melted into the recesses of the printed bench top model using a soldering iron. The face mask was casted with 2k type silicone (www.silikonfabrik.de, Ahrensburg, Germany) for 12 h, the mask could then be removed. Before insertion, it was trimmed using scissors (Fig. 2).

### Study conduction and study participants

**Fig. 3:**
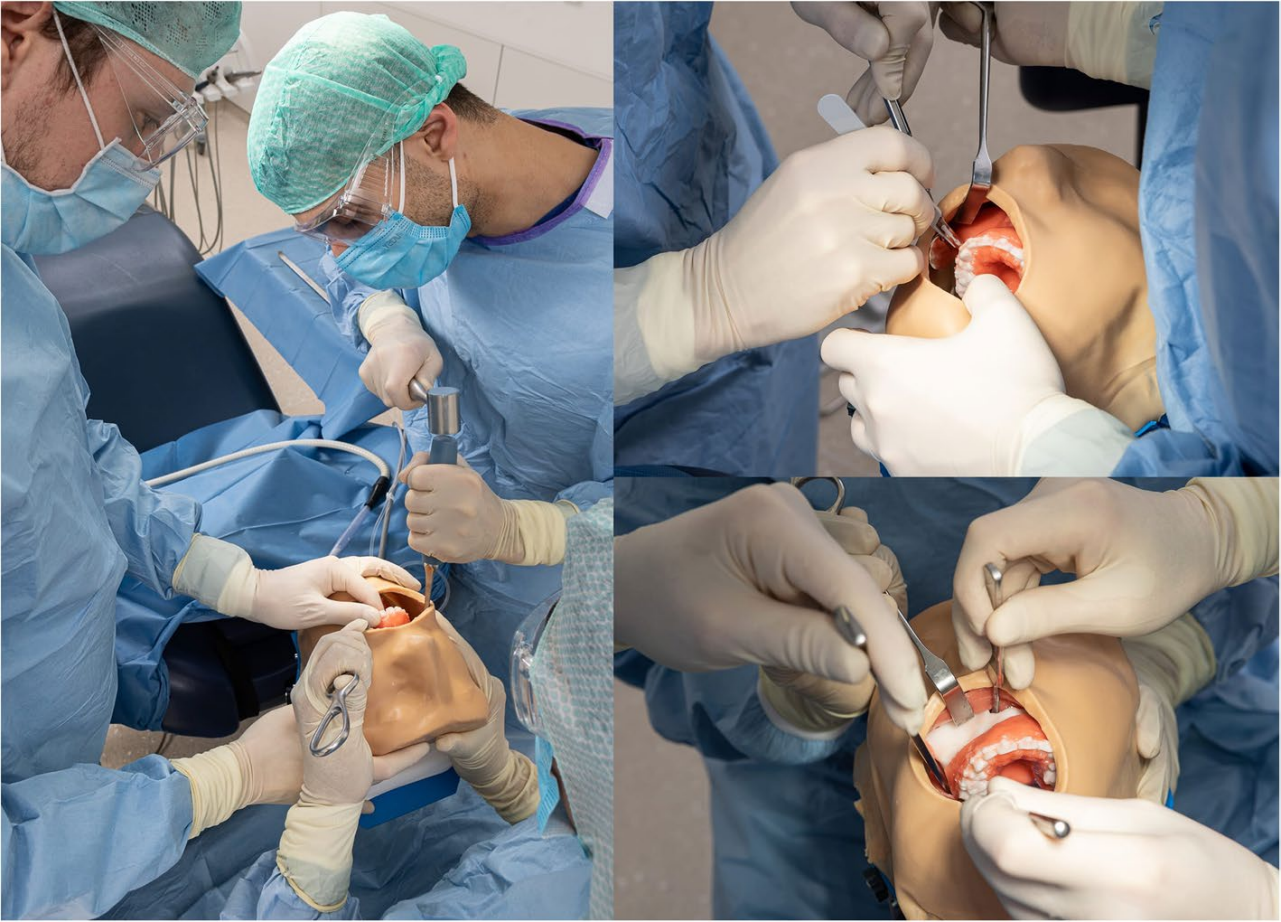
Testing of the 3D-printed simulator under realistic conditions by two experienced orthognathic surgeons

**Fig. 4:**
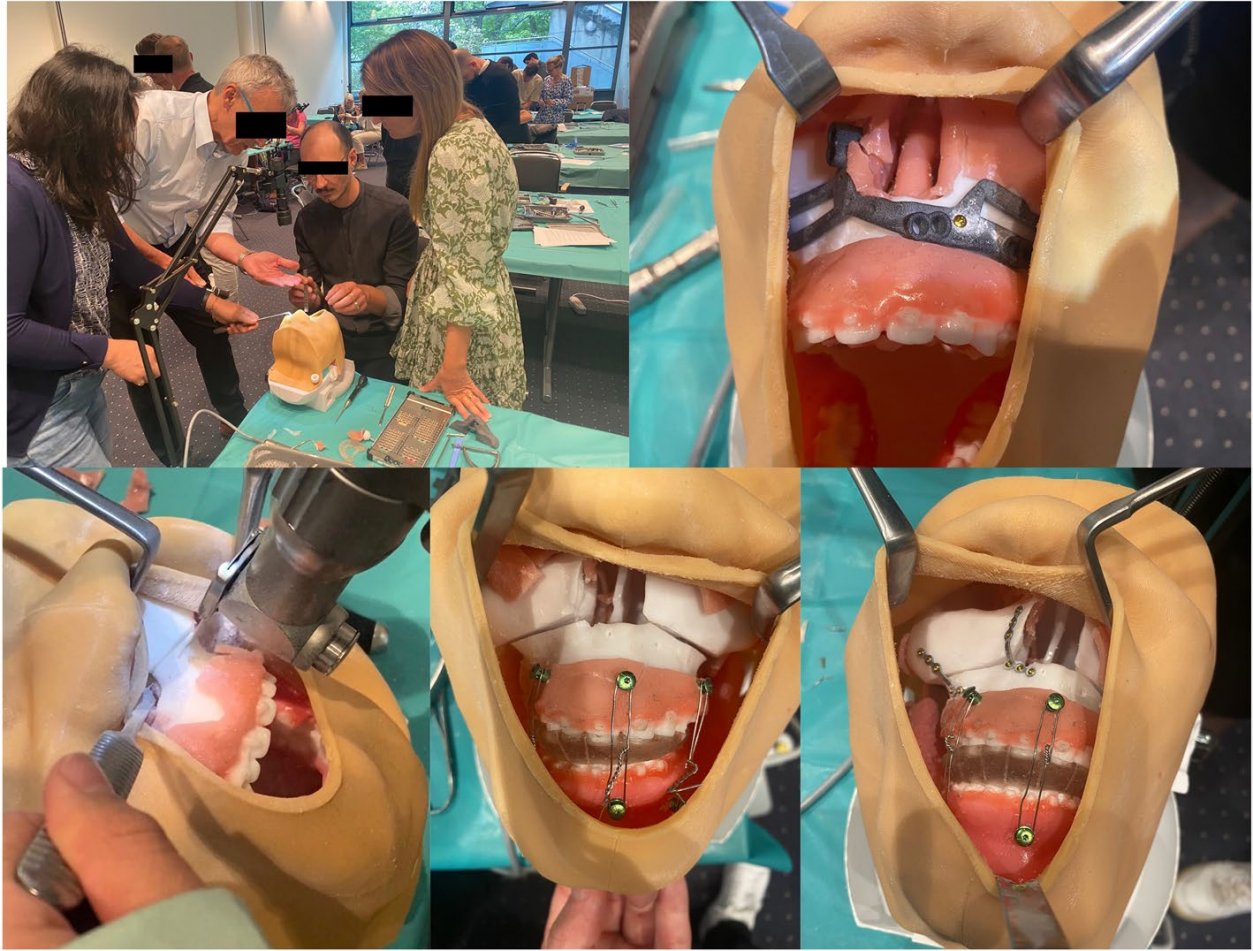
Surgical residents perform a bimaxillary osteotomy on the 3D-printed simulator under guidance of experts within two orthognathic surgery workshops organized by the Young Forum of the German Society of Oral and Maxillofacial Surgery

Prior to its utilization in the planned study, a prototype simulator was developed and assessed by two experienced maxillofacial surgeons affiliated with the Department of Oral, Maxillofacial, and Facial Plastic Surgery, University Hospital Frankfurt, Frankfurt, Germany (Fig. 3). These surgeons possessed substantial experience, each having performed over 70 bimaxillary osteotomies. They were requested to execute both a LeFort I and a Bilateral Sagittal Split Osteotomy (BSSO) on the simulator to gauge its suitability and provide feedback for enhancements. This preliminary evaluation exposed deficiencies in the handling of the model, which were subsequently rectified. For instance, the initially intended method of mounting the bench top phantom head (Fig. 2) was substituted with table mounting phantom head (Fig. 4).

The study participants consisted of resident Oral and Maxillofacial surgeons who attended two courses on orthognathic surgery organized by the Young Forum [24] of the German Society of Oral and Maxillofacial Surgery during two conferences in 2023. The course commenced with a theoretical lecture covering the historical progression of orthognathic surgery and a systematic guide on conducting a LeFort I osteotomy and a bilateral sagittal split osteotomy following the Hunsuck/Epker approach. Following the theoretical session, participants carried out a bimaxillary osteotomy (comprising LeFort I and BSSO procedures) under the guidance of the lecturers (Fig. 4). To ensure each resident had the opportunity to perform at least one LeFort I osteotomy and one BSSO, two residents shared one simulator. Necessary instruments, osteosynthesis materials, equipment and material costs for the procedures were generously provided by KLS Martin (KLS Martin GmbH, Tuttlingen, Germany) and Medartis (Medartis AG, Basel, Switzerland) at no cost. The course spanned a duration of 240 min.

Participation in the study was entirely voluntary and was initiated only after obtaining written informed consent from the participants, with the option to withdraw consent at any point. Fundamental data concerning participants’ age, gender, and residency duration were collected via a questionnaire.

### Evaluation of the models

At the end of the course, participants were asked to fill out a questionnaire, which was a translation into German and adaptation of a previously used and published questionaire [18] (Table 1). A native English speaker translated the questionnaire into German. In this questionnaire, each statement could be rated on a Likert scale ranging from 1 = “does not apply at all” to 10 = “fully applies”. Moreover, the questionnaire left room for free-text commentaries and suggestions for improvements at the end.

**Table 1:** Results of the evaluation regarding simulation reality (*AVG* average, *MED* median, *SD* standard deviation, *n* number of participants)

Item	AVG	MED	SD	*n*
The surgical simulator is realistic	7.16	7	2.03	31
The anatomical representation of each component is realistic	7.10	7	2.04	31
The haptical feedback of the soft tissues is realistic	4.52	4	2.66	31
The haptical feedback of the hard tissues ist realistic	6.74	7	2.49	31
The freedom of movement when using the istruments is realistic	7.58	8	2.04	31
The simulator is a useful instrument to train the soft tissue management	6.68	7	2.89	31
The simulator is a useful instrument to train the LeFort I osteotomy	8.26	9	2.14	31
The simulator is a useful instrument to train the BSSO	8.16	9	2.08	31
The simulator is a useful instrument to train the osteosynthesis	8.19	9	2.09	31
The simulator is a useful instrument to learn each step of a bimaxillary osteotomy	8.58	9	1.64	31
I like on the simulator that: - Realistic representation of human anatomy (*n* = 10) - Realistic bone simulation (*n* = 3) - It enables a structured training of the surgical steps (*n* = 5) - Realistic and structured process (*n* = 4)				
The simulator could be improved by: - More elastic soft tissue simulation (*n* = 8) - Anterior sinus bone to thick (*n* = 2)				

### Cost analysis

For the cost analysis, it was assumed that a student assistant was employed at a rate of €15 per hour. This covered tasks such as the development of printable files, 3D printing, and post-processing of the models. Additionally, 15 h of design and development work were included and distributed evenly across ten simulators to reflect a realistic per-unit allocation. Material costs were calculated based on official retail prices (as of 2022), although some components—particularly resin materials—were obtained at discounted rates. The acquisition costs of the 3D printers used were proportionally included in the overall calculation. Several components, such as the table mount, silicone face mask and ball-joint magnets, were designed for repeated use and do not require replacement after each use. To ensure uninterrupted workflow during hands-on workshops, multiple sets of these reusable parts were manufactured in advance. The amount of material required for each component was estimated using Chitubox Slicer (version 1.6.3, Chitubox, Guangdong, China) for resin-based parts and Prusa Slicer (Prusa Research a.s., Czech Republic) for filament-based parts.

### Data analysis

Microsoft Excel 2016 for Mac (version 15.24, Portland, OR) and Graphpad Prism (version 6.0 for Mac, Graphpad Software, La Jolla California USA) were used to conduct the statistical evaluation and graphical display of the data set. For the descriptive analysis of the data set, descriptive data such as the arithmetic mean, minimum value, maximum value, the median and the standard deviation (SD) were calculated.

## Results

A total of 31 residents specializing in Oral and Maxillofacial Surgery (OMFS) took part in the study. Out of these participants, ten were females, while twenty-one were males. The average age of the participants was 32.17 years, with a standard deviation of 3.53 years. Most of the participants were in their third year of a 5-year training program aimed at achieving board certification in Oral and Maxillofacial Surgery. On average, each participant had performed 5.57 bimaxillary osteotomies in the past, with a standard deviation of 13.35.

The overall consensus among participants was that the surgical simulation involving the 3D-printed simulator was considered realistic (Table 1). On average, the simulator received a rating of 7.16, with a standard deviation of 2.03. Specifically, participants found the anatomical representation (average rating of 7.10, SD = 2.04), the haptic feedback related to hard tissue (average rating of 6.74, SD = 2.49), and the limited range of motion while operating in the oral cavity (average rating of 7.58, SD = 2.04) provided by the 3D-printed simulator to be realistic.

Furthermore, the 3D-printed simulator proved to be a valuable tool for training in various surgical techniques. It was found to be effective when training the LeFort I osteotomy (average rating of 8.26, SD = 2.14), the Bilateral Sagittal Split Osteotomy (BSSO) (average rating of 8.16, SD = 2.08), the osteosynthesis of bony segments (average rating of 8.19, SD = 2.09), and the operational steps of a bimaxillary osteotomy (average rating of 8.58, SD = 1.64).

However, participants generally did not find the haptic feedback provided by the gingival mask to be particularly realistic, giving it an average rating of 4.51, with a standard deviation of 2.66.

In the free-text comments, participants notably commended the 3D-printed simulator for its accurate representation of human anatomy (mentioned by 10 participants) and its ability to facilitate structured simulations of bimaxillary osteotomies (mentioned by 5 participants). Suggestions for improvement mainly centered around the hardness of the gingival mask, with many participants expressing that the material was too rigid (mentioned by 8 participants).

The total cost of producing a 3D-printed simulator mounted up to 181.55 Euro (Table 2).

**Table 2:** Overview of the production costs for one 3D-printed simulator

Component	Unit cost (€)	Quantity used	Total cost (€)
Table mount (470 g PLA)	29.99	0.47	14.10
Screw handles (16 g PLA)	29.99	0.016	0.48
Mold for silicone mask (1558 g PLA)	29.99	1.558	46.72
Maxilla gingiva (80 ml Resione F80)	139.99	0.08	11.20
Mandible gingiva (70 ml Resione F80)	139.99	0.07	9.80
Maxilla bone model (280 ml standard resin)	31.0	0.28	8.68
Mandible bone model (100 ml standard resin)	31.0	0.1	3.10
Ball joint magnets (2 pieces)	10.89	2.0	21.78
Threaded rod (4 cm)	1.59	0.04	0.06
Isopropanol (100 ml)	12.95	0.1	1.29
Thread inserts (5 pieces)	0.3	5.0	1.50
Screws (5 pieces)	0.16	5.0	0.80
Silicone (300 ml)	29.75	0.3	8.92
Release spray (50 ml)	11.75	0.05	0.59
Silicone dye (3 g)	7.75	0.003	0.02
Labor (printing + post-processing, €15/h)	15.0	2.0	30.00
Design development (15 h @ €15/h ÷ 10 simulators)	15.0	1.5	22.50
Total cost per simulator			**181.55**

## Discussion

The present study describes the development and application of an innovative surgical simulator for orthognathic surgery. The motivation for development arose from the observation that the surgical training, both quantitatively and qualitatively, varies significantly on a nationwide scale. The reasons for this variation are manifold and range from staffing shortages and limited surgical capacities to objective agreements and unstructured internal continuing education curricula. Advancements in the field of 3DP technology are continually opening up more innovative opportunities in the development of surgical simulators[18, 19].

Unlike previous models by Bertin et al. and Yoshida et al., our simulator incorporates multiple innovations: (1) realistic articulation of the maxilla and mandible via magnetic ball joints, (2) intraoral space restriction through a realistic silicone face mask and (3) a soft-tissue layer to simulate mucosa. Although no direct comparison was made, these additions represent a significant technical advancement. Quantitative comparisons with existing simulators are planned for future studies.

The 3D-printed simulator presented in this study im summary was regarded as a realistic and proved valuable for training in specific surgical techniques such as LeFort I osteotomy, BSSO, osteosynthesis, and bimaxillary osteotomy. However, the gingival mask’s haptic feedback left room for improvement. The low rating for soft tissue haptic feedback (4.51/10) highlights the need for material innovation. One proposed solution is the use of multi-material printing or integration of advanced silicone blends that better replicate the viscoelastic properties of human soft tissue. Ongoing work is focused on developing such materials to enhance realism.

While VR simulators offer immersive visual training, they often lack tactile feedback and require costly equipment, limiting accessibility. Our approach emphasizes physical simulation with reusable, low-cost components. This makes it particularly suitable for broader dissemination, especially in resource-limited settings.

Cadaveric training continues to be an invaluable asset for surgical education, offering unparalleled opportunities for anatomical exploration and tactile exposure to diverse tissues. However, the declining availability of body donors poses a significant limitation. In contrast, animal models serve as a viable alternative, albeit subject to ethical considerations [25]. Nonetheless, the utility of animal models in acquiring surgical skills is deemed both meaningful and necessary by participants from various disciplines [26, 27]. With the enhanced availability of 3DP technology and the development of innovative printing materials, the rapid and relatively cost-effective fabrication of individualized training models has been integrated into the practical, model-based hands-on training for both medical students and residents [18, 28]. The primary challenge in this context has consistently been the accurate reproduction of anatomical structures with corresponding tactile fidelity. The viscoelastic characteristics of tissues, including hysteresis, creep, and stress relaxation, are attributable to the presence of biopolymers as well as intracellular and extracellular fluids. Consequently, the replication of specific anatomical structures may necessitate the utilization of multiple materials within each tissue type [29]. This challenge is underscored in the current study by the relatively low scores attributed to the haptic properties of the mucosal tissue.

A general limitation common to all surgical models—whether cadaveric, animal-based, or 3D-printed—is the frequent absence of quantifiable outcomes. Hence, commonly, the focus of evaluations lies on comparing two types of training models or entities, rather than assessing the model-dependent improvement in surgical skills when applied in a real-world operating room. This can be viewed as a limitation of this study. A prospective study design with pre- and post-training skill assessments would be necessary to objectively quantify skill acquisition. Future studies will aim to incorporate such objective metrics, including performance-based assessment tools.

Emerging approaches are exploring the utilization of virtual reality, mixed reality, and augmented reality devices through various apparatuses such as head-up displays or glasses. Although new systems incorporating haptic modules do exist, the sense of authenticity is often compromised due to the primary focus on visual experience; haptic feedback and physical interaction experiences remain suboptimal [30, 31]. A general limitation common to all surgical models—whether cadaveric, animal-based, or 3D-printed—is the frequent absence of quantifiable outcomes hence, commonly, the focus of evaluations lies on comparing two types of training models or entities, rather than assessing the model-dependent improvement in surgical skills when applied in a real world operating room, which can be viewed as a limitation of this study.

The 3D-printed model in this study is the first surgical simulator for the training of orthognathic surgery that takes the soft tissue situation into account.

Another aspect currently lacking in the simulator is the integration of neurovascular structures, especially the inferior alveolar nerve. Since nerve preservation is critical in orthognathic surgery, future iterations of the simulator may benefit from embedding a nerve pathway using colored silicone or conductive materials to provide a tactile cue for its identification and protection.

In addition, the limited space of movement in the oral cavity was realistically depicted with a face mask and the position and freedom of movement of the lower jaw compared to the upper jaw was also considered during the simulator planning and production. Even though the soft tissue haptic feedback was rated poorly, this is already a significant improvement compared to the previously published studies, which do not include parameters such as soft tissue and the intraoperative restriction of the surgeon’s freedom of movement in the simulation [20, 21]. It can therefore be assumed that if the innovative simulator used here was directly compared with one of the previously developed ones, the assessment of the soft tissue haptics and the overall rating would have been significantly better. However, this shows a limitation of the study, which did not evaluate the new surgical simulator for the training of orthognathic surgery against another one. On the other hand, the simulators used previously, which basically only show the bony situation of individual jaws, are not comparable to the one used in this study. The simulator in this study was assessed by two very experienced maxillofacial surgeons and the course participants also had a relatively high level of experience with an average of six bimaxillary osteotomies performed, too, which means that the evaluation results of this study are also based on a certain comparison of the simulator with the real patient situation. In contrast, the study by Yoshida et al. [21] was conducted with dental students and in the study by Bertin et al. [20] oral and maxillofacial surgery residents were trained, but the number of dysgnathic surgery procedures performed by the residents was not recorded. In addition to the improved reproduction of anatomical structures, the cost-effectiveness of the simulators can also be assessed as very high, as they do not cost more than the previous models (US$155) despite the soft tissue and jaw joint components [20]. While the total cost of € 181.55 was carefully itemized, durability testing over multiple uses is ongoing. Preliminary use in two workshops indicates that key components, including the phantom head and joint system, remain intact after repeated use. A direct cost comparison with the Bertin model (€155) supports our claim of high cost-efficiency despite enhanced features.

To make the simulator even more realistic, besides the improvements in simulating soft tissue, the integration of a soft tissue inferior alveolar nerve simulation would be conceivable, since the intraoperative handling and protection of this structure can have a decisive influence on the patient’s postoperative quality of life.

## Conclusion

Although the acquisition of actual surgical skills is ultimately achieved through human-based practice, the use of surgical simulators presents a sustainable, innovative and financially viable approach to standardize and continually enhance the surgical education of aspiring surgeons. The present study introduces, for the first time, a realistic surgical simulator for training in orthognathic surgery. Noteworthy aspects include the cost-effective production and the reusability of many simulator components.

## Data Availability

No datasets were generated or analysed during the current study.

## Abbreviations

3DP: Threedimensional printing
BSSO: Bilateral sagittal split osteotomy
